# Efficacy of digital educational intervention using I-Change model in promoting preventive behaviors for cervical cancer among Iranian women: A randomized controlled trial

**DOI:** 10.34172/hpp.025.43722

**Published:** 2025-05-06

**Authors:** Sara Kazemi, Fatemeh Zarei, Alireza Hidarnia, Fatemeh Alhani

**Affiliations:** ^1^Department of Health Education and Health Promotion, Faculty of Medical Sciences, Tarbiat Modares University (TMU), Tehran, Iran; ^2^Department of Nursing, Faculty of Medical Sciences, Tarbiat Modares University (TMU), Tehran, Iran

**Keywords:** Health behavior, Health education, Randomized controlled trial, Uterine cervical neoplasms

## Abstract

**Background:**

Cervical cancer (CC) is a major health concern for women, yet stigma and embarrassment often prevent preventive care. Digital health education offers a private, accessible way to learn about CC prevention. This study evaluated the effectiveness of a digital educational intervention based on the I-Change model in promoting preventive behaviors among Iranian women.

**Methods:**

A randomized controlled trial (RCT) was conducted with 210 women (18–49 years) from Ramsar, Iran. Participants were assigned to three groups: two intervention groups (mobile app and digital booklet) and a control group. The "Evaluation of Preventive Behaviors Against CC (PERCICA)" questionnaire assessed outcomes. The intervention included a pre-test, digital education, and follow-ups immediately and at 12 weeks post-intervention. Data were analyzed using t-tests, analysis of Variance (ANOVA), analysis of covariance (ANCOVA), and repeated measures ANOVA.

**Results:**

The app group showed significant improvements in knowledge, perceived risk, self-efficacy, cognitive barriers, and social support (*P*<0.05). Preventive behaviors (e.g., condom use, Pap tests, genital exams) were highest in the app group (67.12%, 95% CI: [29.32±2.35]), followed by the booklet group (38.57%, 95% CI: [9.55±2.17]).

**Conclusion:**

The app was more effective in sustaining behavioral changes and promoting CC prevention than the booklet, highlighting the value of tailored digital education for sensitive health topics.

**Trial Registration:**

ClinicalTrials.gov IRCT20181205041861N3. Registered V2.0 on 26 October 2021 with the IRCTID, V1.0. https://irct.behdasht.gov.ir/trial/57157.

## Introduction

 Women’s health plays a crucial role in the overall health and development of societies. Women’s health plays a vital role in shaping the well-being of children, families, and communities.^1^ Cervical cancer (CC), in particular, is a pressing public health issue due to its high morbidity and mortality, coupled with the preventable through early screening. Globally, CC is the fourth most common cancer among women and one of the leading causes of cancer-related deaths in developing countries.^2^ CC accounted for 341 831 deaths worldwide in 2022.^3^ Approximately half a million new cases of CC are diagnosed each year, with the majority occurring in low- and middle-income countries.^4,5^ Literature suggests that over 90% of global CC deaths occur in these regions, with Asia accounting for 144,400 deaths annually.^6^ Persistent infection with high-risk human papillomavirus (hrHPV) is the leading cause, contributing to nearly 99% of CC cases.^7^ Other risk factors include smoking, a history of sexually transmitted diseases, and low socioeconomic status.^8,9^

 CC ranks among the five most common cancers and is the fourth leading cancer diagnosed in females in Iran. Late-stage diagnoses and high mortality rates persist, with HPV-16 identified as the most prevalent strain associated with CC in the country.^10^ Alarmingly, CC incidence is rising among younger women with the mortality-to-incidence ratio reported to be 42%.^11^ Lack of awareness about preventive measures like Pap smears is a major barrier, influenced by cultural, economic, and social factors.^12^ Although the Pap smear test is available free of charge in many Iranian health centers, the uptake remains low, with only 14.8% to 28.3% of eligible women undergoing the test.^13^ Contributing factors include fear of pain, embarrassment, and low perceived risk.^13-16^ Neglect, time constraints, financial limitations, and lack of partner encouragement further hinder screening efforts.^17^ CC can lead to severe health complications, particularly affecting women of reproductive age, and can spread to nearby organs, exacerbating health outcomes.^18^ Despite these challenges, CC is largely preventable, and its incidence can be dramatically reduced through targeted education, screening, and timely intervention.^19^ Early detection through regular Pap smears is crucial. Yet the sociocultural stigma around sexual health education remains a significant barrier.^20^ Resistance to receiving sexual health information may drive individuals to seek unreliable sources, increasing misinformation.^21^

 Mobile health (m-Health) technology offers a promising solution to these challenges, providing a safe, private, and accessible platform for health education. Self-paced learning via mobile applications is particularly appealing to younger generations and can mitigate the embarrassment associated with face-to-face consultations.^22^ m-Health has gained momentum globally; as it empowers individuals to manage their health, reduces healthcare inequities, and enhances cost-effectiveness.^23-25^ In the context of CC prevention, mobile apps and online platforms can facilitate early detection and improve health outcomes.^26^ With Iran ranking 12th globally in smartphone penetration in 2019 and 70% of Iranians owning smartphones, digital education could serve as an effective alternative to traditional health education methods.^27^ However, many cancer-related apps lack quality and scientific validity^28,29^ To address these challenges and promote participation in CC screening, behavioral change theories are essential. This study adopts the I-Change model, an integrative framework that builds on the Attitude-Social Influence-Self-efficacy (ASE) model and incorporates additional components like social support and modeling.^30^ Given the increasing burden of CC in Iran, this study aims to assess the efficacy of a digital health educational intervention based on the I-Change model in promoting preventive behaviors for CC among Iranian women.

## Material and Methods

###  Study design and participants

 This study was a randomized controlled trial (RCT) conducted among 210 Iranian women recruited from hospitals and gynecological clinics in Ramsar, located in the north of Iran, in 2024. Participants met the following inclusion criteria: women aged 18–49 years; married (with at least one sexual relationship experience); residing in Northern Iran during the study period; willing to participate and provide informed consent; possessing an Android-based smartphone and being able to use it; and having no mental health disorders, drug dependency, or addiction. Exclusion criteria included missing over two training sessions, having a medical condition that prevented participation, or losing interest in the study.^31^ The sample size was determined based on power analysis and previous studies, ensuring sufficient statistical power to detect meaningful differences between the groups. Details of the sample size calculation are provided in the following section.

###  Sample size 

 The sample size was calculated based on a prior study. If the difference in the mean knowledge score between the intervention groups before and after the intervention was expected to be 0.53 units, with a pre-intervention standard deviation of 1.91 and a post-intervention standard deviation of 2.01, and with a significance level of 95% (α = 0.05) and a power of 90% (β = 0.10), it was determined that approximately 70 participants per group would be needed, accounting for a potential dropout rate of 15%.

###  Recruitment process

**Figure 1:**
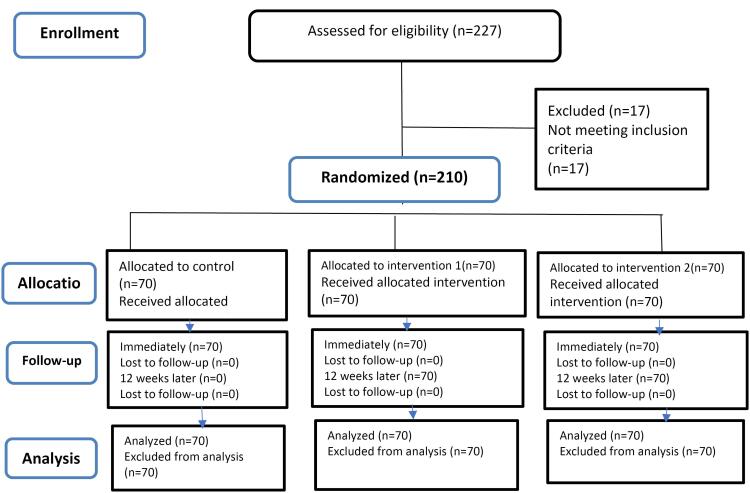
CONSORT flowchart of the study

 In the initial phase, after coordination with affiliated centers, a list of 1,130 eligible women who had visited the hospitals and gynecology clinics in the previous six months was compiled. Potential participants were invited via digital platforms, including Instagram, WhatsApp, and Telegram. Of the 227 women who expressed interest, 210 met the eligibility criteria after screening, as 17 individuals were unable to use the Android app required for the study. Before giving their informed consent, all participants were provided with detailed information about the study. Due to the nature of the intervention, blinding of participants and study staff was not feasible. The randomization process and sample allocation are depicted in the CONSORT flowchart (Figure 1).

###  Randomization

 After obtaining informed consent and collecting baseline data, participants were randomly assigned to either the intervention or control group using a restricted block randomization design. Each participant was given a unique research identification number. The participants were assigned to one of three groups in a 1:1:1 ratio using an online randomization tool provided by https://www.sealedenvelope.com/simple-randomiser/v1/lists. Group A was designated as intervention training via Mobile App, group B as intervention training via Digital Booklet, and group C as the control group. The random allocation continued until the target sample size was reached. Intervention effectiveness was assessed at two post-test time points: immediately and 12 weeks after the intervention. The study spanned 16 weeks from December 22, 2023, to mid-April 2024

###  Educational content development for mobile application and digital booklet using the I-Change model

 An I-Change Model based educational intervention was developed, which served as the theoretical framework to promote preventive behaviors related to CC. The content was tailored through a literature review, findings from the qualitative phase of the study, and pre-test results. The instructional goals were developed according to the I-Change constructs, addressing the educational needs and learning challenges faced by Iranian women regarding CC. To ensure culturally and contextually relevant content, the research team evaluated CC prevention education practices in Iran through a literature review and qualitative research. The educational content was categorized into five domains: knowledge, perceived risks, perceived cognitive barriers, preventive self-efficacy, and perceived social support. Guidelines from credible sources such as the World Health Organization (WHO),^3,32^ the Centers for Disease Control and Prevention (CDC),^33,34^ and the Iranian Ministry of Health,^35,36^ were analyzed to shape the content. The final material covered essential topics related to CC, including symptoms, diagnostic and treatment methods, and prevention strategies. It also included methods for enhancing self-efficacy, perceived vulnerability, and social support to facilitate preventive behaviors (Table 1).

**Table 1:** Domains, instructional goals, subordinate skills, behavioral objectives, and educational content

**Domain**	**Instructional goals**	**Subordinate skills**	**Behavioral objectives**	**Educational content**
Knowledge	Enhance women's knowledge of CCP	- Understand how CC occurs. - Recognize CCP procedures. - Identify when, where, and by whom to be examined. - Know the risks associated with multiple sexual partners and HPV.	- Describe the symptoms and complications of HPV. - Outline diagnostic methods for HPV. - Explain CC prevention methods, routes, and risk behaviors.	- Types, symptoms, and complications of CC. - Diagnostic and treatment methods. - CCP routes and risk behaviors.
Perceived risks	Cultivate awareness of perceived risks for CC prevention	- Understand personal risk. - Recognize the potential for developing CC.	- Identify transmission pathways of CC.	- Ways of transmission and associated risk factors for CC.
Perceived barriers	Acknowledge barriers to CCP	- Recognize the barriers preventing preventive behaviors, such as fear, embarrassment, and guilt.	- Identify types of barriers to CCP. - Develop strategies to reduce or eliminate these barriers.	- Types of barriers to CCP. - Strategies to overcome fear, embarrassment, and guilt.
Preventive self-efficacy	Strengthen confidence in preventive actions for CCP	- Gain confidence in using preventive methods like condoms and Pap smears. - Understand CC risk factors and coping skills.	- Demonstrate how to perform a Pap smear and describe its benefits. - Outline risk factors and prevention strategies for CC.	- Pap smear demonstration. - Benefits of Pap smear. - CC-related risk factors. - Coping strategies for those diagnosed with CC.
Perceived social support	Build awareness of social support for CCP	- Understand the benefits of counseling and accessing information about CCP through social support.	- Recognize the importance of friends, family, healthcare providers, and various media in promoting CC prevention behaviors.	- Accessing CCP information via friends, doctors, midwives, healthcare staff, books, magazines, media, and the internet.

CC, cervical cancer; CCP, cervical cancer prevention;

###  Mobile application and digital booklet

 The educational content was delivered through two different platforms: a mobile application (PERCICA) and a digital booklet. Both formats followed the same structure, with the primary difference being the medium of presentation. The PERCICA app, designed specifically for this study, featured an intuitive interface, multimedia elements (e.g., short films, motion graphics, podcasts, games, and animations), and culturally tailored educational material. It was developed in Java with a focus on user experience (UX). In contrast, the digital booklet presented the same information in an accessible, text-based format, complemented by visuals to maintain engagement. Both formats adhered to the constructs of the I-Change Model, ensuring consistency in educational delivery while offering flexibility based on user preferences.

###  Evaluation of the PERCICA app and digital booklet

 The educational material was pilot-tested with 20 women. These women provided feedback on the relevance, comprehensibility, simplicity, and appeal of the content through a 14-item questionnaire distributed via WhatsApp. The feedback was then used to refine the materials, which were subsequently incorporated into the final version of the app for the intervention.

###  Intervention implementation

**Figure 2:**
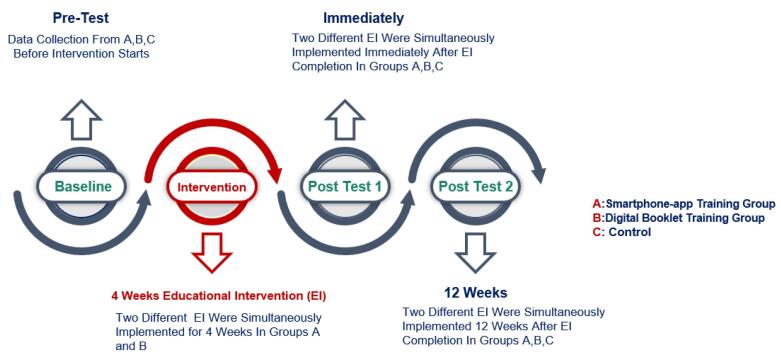
Intervention timeline

 Participants in the PERCICA app group, and digital booklet group received the educational intervention through the PERCICA app, following a four-week training program. The app group was provided tutorial videos for installation and usage, while the booklet group received PDF files accessible on their smartphones. Participants could contact the researchers for technical support throughout the study. The control group did not receive any formal training during the study period but was granted access to the app after the final post-test. Participants were also offered a voucher for a free CC screening at a midwifery clinic as a token of appreciation. Intervention timeline illustrated in Figure 2.

###  Instruments for data collection

 The PERCICA questionnaire, based on the I-Change model, was used to assess preventive behaviors. The questionnaire consisted of two sections: demographic information (age, education, occupation, economic status, marital status) and 28 items across five dimensions: knowledge (4 items), preventive self-efficacy (4 items), perceived cognitive barriers (12 items), perceived social support (4 items), and perceived risks (4 items). Knowledge was assessed with a three-point scale (correct, incorrect, don’t know), while the remaining dimensions were measured on a five-point Likert scale (strongly disagree to strongly agree). The reliability of the questionnaire was supported by Cronbach’s alpha, which ranged from 0.700 to 0.907. Additionally, the intraclass correlation coefficient (ICC) was found to be 0.919. The total score was calculated by summing all item scores, with higher scores reflecting better preventive behaviors.

###  Statistical analysis 

 Data were analyzed using IBM SPSS Statistics Version 26 (IBM Corp., Armonk, NY, USA). The chi-square test, Fisher’s exact test, and independent t-tests were used to compare socio-demographic characteristics between groups. Differences in mean scores for knowledge, preventive self-efficacy, perceived cognitive barriers, perceived social support, and perceived risks between the three groups before and after the intervention were analyzed using independent t-tests, analysis of Variance (ANOVA), and analysis of covariance (ANCOVA). ANCOVA was chosen to adjust for potential confounders and baseline differences in outcome variables, thereby improving the precision of the treatment effect estimates. Confounders such as baseline scores of dependent variables and significant socio-demographic variables were adjusted for in the model. Repeated measures ANOVA (RMANOVA) was used to compare changes in mean scores within each group over time. Before conducting ANOVA, ANCOVA, and RMANOVA, key assumptions—including normality (Shapiro-Wilk test), homogeneity of variances (Levene’s test), and sphericity (Mauchly’s test for RMANOVA)—were assessed. In cases where assumptions were violated, appropriate remedies were applied. If normality was not met, data transformation or non-parametric alternatives were considered. In case of heterogeneity of variances, Welch’s ANOVA was used, and if the sphericity assumption was violated in RMANOVA, the Greenhouse-Geisser correction was applied. Post-hoc analysis with Bonferroni correction was performed where applicable. A *p*-value of less than 0.05 was considered statistically significant.

## Results

###  Participant recruitment and flow

 A total of 227 women were initially assessed for eligibility. Of these, 17 did not meet the inclusion criteria due to reasons such as lack of smartphone access, low digital literacy, or unwillingness to participate. The remaining 210 participants met the inclusion criteria and were randomized into three groups: mobile app intervention (n = 70), digital booklet intervention (n = 70), and control (n = 70). During the study, none of participants withdrew, leaving 210 participants for the final analysis. The CONSORT flow diagram (Figure 1) provides a detailed overview of the participant recruitment and allocation process.

###  Socio-demographic characteristics

 A total of 210 women aged 18-49 years were randomized to three groups: control, mobile app intervention, and digital booklet intervention (Table 2).

**Table 2:** Comparison of socio-demographic characteristics of the participants between the three groups

**Demographic variables**	**PERCECA app (n=70)** **No. (%)**	**Digital booklet (n=70)** **No. (%)**	**Control (n=70)** **No. (%)**	**Total (N=210)** **No. (%)**	***P*** ** value**
Women’s age (y)					
≤ 25	20 (28.6)	25 (35.7)	20 (28.6)	65 (30.9)	0.153
26–35	10 (14.2)	17 (24.3)	19 (27.1)	46 (21.9)	
36–45	25 (35.7)	13 (18.5)	20 (28.5)	58 (27.6)	
≥ 46	15 (21.5)	15 (21.5)	11 (15.8)	41 (19.6)	
SD ± Mean	6.90 ± 33.71	7.11 ± 31.29	6.90 ± 33.71	7.42 ± 32.55	
Minimum–Maximum	19-49	18-49	20-49	18-49	
Women’s marital status					
Permanent marriage	40 (57.1)	32 (45.7)	45 (64.3)	117 (55.7)	0.227
Temporary marriage	17 (24.3)	25 (35.7)	15 (21.4)	52 (27.1)	
Divorced	13 (18.6)	13 (18.6)	10 (14.3)	36 (17.1)	
Education level of women					
High school	11 (15.7)	16 (22.9)	14 (20)	41 (19.5)	0.749
Diploma	14 (20)	16 (22.9)	17 (24.3)	47 (22.4)	
Academic	45 (64.3)	38 (44.3)	93 (44.3)	93 (44.3)	
Employment status					
Housewife	25 (35.7)	23 (32.8)	25 (35.7)	73 (34.7)	0.153
Student	20 (28.6)	25 (35.7)	14 (20)	59 (28.1)	
Employee	25 (35.7)	22 (31.5)	31 (44.3)	78 (37.2)	
Economic status					
Very favorable	8 (11.5)	11 (15.7)	5 (7.1)	24 (11.4)	0.271
Favorable	40 (57.1)	36 (51.5)	45 (64.3)	121 (57.6)	
Unfavorable	15 (21.4)	12 (17.1)	15 (21.5)	42 (20)	
Very unfavorable	7 (10)	11 (15.7)	5 (7.1)	23 (11)	

###  Primary outcomes: five measured variables

 The primary aim was to evaluate the effects of the I-Change-based intervention on knowledge, self-efficacy, perceived social support, cognitive barriers, and perceived risks. These variables were measured among women in the intervention and control groups before the intervention, and then again at immediately and 12 weeks post-intervention.

###  Knowledge of CC preventive behaviors 

 ANOVA results showed that there was no significant difference in the mean knowledge scores about CC prevention among the control group, the first intervention group (PERCICA app), and the second intervention group (digital booklet) before the intervention (*P* = 0.135). However, a significant difference was observed immediately after the intervention (*P* < 0.001). Bonferroni pairwise comparisons showed that this difference was significant between the control group and both intervention groups (*P* < 0.001). Both intervention groups had significantly higher knowledge scores than the control group at both time points. Both intervention groups showed significant improvements in knowledge compared to the control group, with no baseline differences. In contrast, the control group’s knowledge scores remained unchanged throughout the study (Table 3).

**Table 3:** The average scores of the five domains before and after the educational intervention in the experimental and control groups

**Variable**	**Pre-test**	**Immediately after intervention**	**12 weeks intervention**	***P*** ** value**
Knowledge				
PERCECA app	3.21 ± 1.51	7.17 ± 1.57	7.47 ± 0.92	< 0.001
Digital booklet	3.15 ± 1.47	7.51 ± 0.94	7.15 ± 1.42	< 0.001
Control	3.25 ± 1.48	3.78 ± 2.09	3.30 ± 1.54	0.591
Perceived risks				
PERCECA app	3.35 ± 1.66	7.21 ± 1.33	7.31 ± 1.37	< 0.001
Digital booklet	4.34 ± 1.62	6.98 ± 1.47	6.35 ± 1.65	< 0.001
Control	3.17 ± 1.55	3.35 ± 1.66	4.35 ± 1.62	0.590
Perceived barriers				
PERCECA app	37.14 ± 5.18	14.21 ± 4.11	14.95 ± 4.18	< 0.001
Digital booklet	41.04 ± 5.21	40.11 ± 5.44	40.08 ± 5.46	< 0.001
Control	39.88 ± 5.19	39.88 ± 5.19	39.88 ± 5.19	0.998
Preventive Self-efficacy				
PERCECA app	11.52 ± 1.81	19.82 ± 0.70	19.77 ± 0.72	< 0.001
Digital booklet	10.42 ± 3.00	10.55 ± 3.02	10.55 ± 3.17	< 0.001
Control	8.85 ± 2.71	8.97 ± 2.57	8.85 ± 2.71	0.456
Perceived Social Support				
PERCECA app	11.78 ± 2.10	19.74 ± 0.89	19.32 ± 1.35	< 0.001
Digital booklet	10.05 ± 2.64	9.90 ± 2.50	9.80 ± 2.62	< 0.001
Control	10.11 ± 2.35	10.11 ± 2.35	10.11 ± 2.35	0.251

*Note*: Data are expressed as mean ± SD.

###  Perceived risks of CC preventive behaviors 

 ANOVA results indicated that There were no baseline differences in perceived risk scores among the groups (*P* = 0.148). According to the ANCOVA test, a significant difference was observed immediately after the intervention (*P* < 0.001). Bonferroni pairwise comparisons showed that this difference was significant between the control group and both intervention groups (*P* < 0.001), but not between the two intervention groups (*P* = 0.990). The mean perceived risk scores in both intervention groups were significantly higher than the control group immediately post-intervention. At three months post-intervention, a similar significant difference was found (*P* < 0.001). However, while the difference between the two intervention groups approached significance (*P* = 0.051), it was not statistically significant. Overall, the perceived risk scores were somewhat higher in the second intervention group before the intervention, but both intervention groups exhibited similar and notable increases immediately and three months after the intervention compared to the control group. This increase appeared to be more sustained in the PERCICA app group (Table 3).

###  Perceived cognitive barriers regarding CC preventive behaviors 

 ANOVA results indicated that there was no significant difference in the mean cognitive barrier scores between the control group, PERCICA app group, and digital booklet group before the intervention (*P* = 0.691). However, based on the ANCOVA test, a significant difference was found immediately after the intervention (*P* < 0.001). Bonferroni pairwise comparisons showed a significant difference between the control group and PERCICA app group (*P* < 0.001), and between PERCICA app group and digital booklet group (*P* < 0.001), but no significant difference between the control and digital booklet group (p = 0.988). Additionally, the ANCOVA test revealed a significant difference between the three groups at three months post-intervention (*P* < 0.001). Bonferroni comparisons showed that this difference was significant between the control and PERCICA app group (*P* < 0.001), as well as between PERCICA app group and digital booklet group (*P* < 0.001). However, no significant difference was found between the control group and digital booklet group (*P* = 1.00). Therefore, the mean cognitive barrier score in PERCICA app group was significantly lower than the other two groups at this time point, with no significant difference between the control and digital booklet group. In conclusion, PERCICA app group demonstrated a substantial reduction in cognitive barriers over time, while both the control group and digital booklet group showed no significant change (Table 3).

###  Preventive self-efficacy regarding CC

 ANOVA results showed that there was no significant difference in the mean preventive self-efficacy scores between the control group, PERCICA app group, and digital booklet group before the intervention (*P* = 0.479). However, according to the ANCOVA test, significant differences were observed immediately and three months post-intervention (*P* < 0.001). Bonferroni pairwise comparisons revealed significant differences between the control group and PERCICA app group (*P* < 0.001), the control group and digital booklet group (*P* < 0.001), and between PERCICA app group and digital booklet group (*P* < 0.001). The mean self-efficacy score in PERCICA app group was significantly higher than both other groups at both time points. Overall, PERCICA app group exhibited a significant increase in preventive self-efficacy over time, while the control group and digital booklet group showed no meaningful change (Table 3).

###  Perceived social support regarding CC preventive behaviors 

 ANCOVA results indicated that there was no significant difference in the mean perceived social support scores between the control group, PERCICA app group, and digital booklet group before the intervention (*P* = 0.221). However, significant differences were observed immediately and three months post-intervention (*P* < 0.001). Bonferroni pairwise comparisons revealed significant differences between the control group and PERCICA app group (*P* < 0.001), and between PERCICA app group and digital booklet group (*P* < 0.001), but no significant difference between the control and digital booklet group. Therefore, PERCICA app group had significantly higher perceived social support scores compared to the other two groups. Overall, the changes in perceived social support scores over time were significantly higher in PERCICA app group, while the control group and digital booklet group showed no substantial change (Table 3).

###  The secondary outcome

 The secondary outcome focused on assessing women’s preventive behaviors toward CC, including condom use, undergoing Pap tests, and having genital examinations at gynecology clinics provided by the research team, arranged free of charge following the intervention period. The overall response rate for engaging in these preventive behaviors was 35.23% (95% CI: [39.88 ± 4.19]). Specifically, the response rate for the smartphone app group was 67.12% (95% CI: [29.32 ± 2.35]), for the digital booklet group it was 38.57% (95% CI: [9.55 ± 2.17]). This highlights the smartphone app’s superior effectiveness in encouraging women to take preventive actions post-intervention.

## Discussion

 This study aimed to evaluate the effectiveness of an I-Change model-based digital educational intervention in enhancing women’s preventive behaviors related to CC.^37-39^ The findings revealed significant differences in CC prevention knowledge scores among the smartphone app group, digital booklet group, and control group following the intervention, aligning with previous studies that emphasize the role of digital health interventions in improving knowledge, attitudes, and preventive behaviors. Participants across all groups were homogeneous in demographic characteristics, and no significant differences were found in baseline knowledge, perceived risk, perceived barriers, self-efficacy, or social support. This study is among the first to apply the I-Change model in a digital educational intervention for CC prevention among Iranian women, demonstrating the effectiveness of mobile health (mHealth) tools in improving preventive behaviors, particularly through smartphone applications and digital booklets. The intervention effectively increased knowledge, reduced perceived cognitive barriers—such as fear—and enhanced self-efficacy, a key determinant in behavior change. Furthermore, it improved perceived social support, which plays a crucial role in reducing stigma and promoting screening uptake. These findings highlight the importance of theory-driven digital health interventions in CC prevention, particularly in resource-limited settings where face-to-face education may be challenging. Supporting our findings, numerous studies highlight that mobile apps and digital booklets effectively enhance perceived risk awareness in various areas such as maternal and child health, diabetes management, and cancer prevention. Increased awareness fosters personal concern about health issues, motivating individuals to adopt preventive measures.^40-42^ These results underscore the importance of targeted interventions in encouraging preventive behaviors. Our study also demonstrated that the intervention significantly reduced perceived cognitive barriers, such as fear, in the smartphone app group post-intervention. This aligns with previous research showing that perceived barriers, including fear, embarrassment, lack of awareness, and logistical challenges like inadequate healthcare access, are critical factors influencing vaccination intent and CC screening.^43,44^ Addressing these barriers is essential for improving early detection and prevention. For instance, a study by Ghalavandi et al similarly reported a reduction in perceived barriers in the intervention group compared to the control group following educational efforts.^45,46^ Differences in perceived barriers—whether physical, material, psychological, or social—may explain variations in preventive behavior adoption. Misconceptions about healthy behaviors must be addressed to facilitate CC prevention. Despite high levels of perceived benefits, behavioral change often remains elusive unless barriers to adopting healthy practices are minimized. Aligning perceived barriers and benefits is crucial for promoting healthy behaviors, as highlighted by Dillard, who emphasized that overcoming barriers is key to initiating behavior change. The educational intervention in this study effectively increased preventive self-efficacy in the mobile app group. Similar studies have supported this finding, highlighting the importance of self-efficacy in promoting sexual health and preventing CC.^47-49^ Women who are self-taught can overcome barriers to receiving screening because their high self-efficacy stems from their confidence in their ability to undergo CC screening.^50,51^ Bandura and Adams identify self-efficacy as a key predictor of behavioral change in high-risk situations, and numerous studies emphasize its correlation with preventive behaviors for CC.^52^ Various forms of self-efficacy, such as the ability to refuse risky sexual behaviors and confidence in undergoing a pap smear, are vital for increasing CC prevention intent. Self-efficacy is a critical concept for health promotion and plays a significant role in preventing CC within key populations. Generally, self-efficacy acts as a mediator between learning and health behaviors, representing an individual’s belief in their ability to successfully adopt health behaviors. The educational content, delivered through a mobile app tailored to the learning needs of these women, appears to have significantly contributed to increased self-efficacy in this study. The designed educational intervention also effectively enhanced perceived social support related to CC among participants. Based on the theory of perceived social support, supportive interactions, along with perceived social support, can protect women from the health consequences of stress, increase adherence to treatment, and promote recovery.^50-52^ Participants in the mobile app group were not limited by the time, location, or geographical restrictions often faced by face-to-face groups. They could exchange messages anytime, offering diverse experiences, perspectives, and resources. Increased knowledge about CC seems to have led to an improvement in perceived social support, reducing both internal and external stigma associated with the disease. This result isconsistent with previous research findings.^23,53^ Positive social support can amplify the social and environmental impact, making pap smear screening more acceptable, promoting screening encouragement, and improving access to healthcare services. Perceived social support encompasses emotional and practical assistance that individuals feel or give to themselves. Through this program, women could gain a broader perspective, which helped alleviate negative feelings and reduced perceived barriers, while improving social support and self-esteem. Social support, through interpersonal exchanges, is one of the most important benefits of online health activities.

## Strengths and limitations, potential biases

 This study successfully developed and implemented an educational intervention to enhance awareness of CC risks among Iranian women, using a mobile app as the primary delivery method. However, the study has some limitations. Participation was limited to women with high digital literacy and smartphone access, which may restrict the generalizability of the findings. As a result, the study’s applicability to women with lower digital health literacy, limited access to technology, or different socioeconomic backgrounds remains uncertain. Future studies should aim to include participants with more diverse demographic and technological backgrounds to gain a broader perspective on the intervention’s impact. Additionally, cultural taboos surrounding CC in Iran present a challenge. Future research should explore how cultural values and beliefs shape women’s attitudes and behaviors toward CC prevention, tailoring interventions to address these sensitivities. Potential biases, such as reliance on self-reported data and limited sample representativeness, were acknowledged in this study. Strategies to mitigate these biases were considered and discussed. Notwithstanding these drawbacks, the results offer insightful information about how well digital health interventions work to prevent CC, especially for populations with mobile technology access. To ascertain whether comparable interventions would produce comparable outcomes in various cultural and healthcare contexts, more research is necessary. Furthermore, the potential of technology-based interventions in educating women about CC prevention remains an area for future investigation.

###  Implications for clinical practice 

 The findings of this study suggest that the educational intervention we developed holds promise for improving CC prevention, as well as the overall sexual health and well-being of women in Iran and similar contexts. The mobile app, designed using the I-Change model, proved effective in increasing knowledge and promoting preventive behaviors related to CC. It also helped reduce barriers associated with the topic, such as fear and stigma. These results suggest that such interventions can serve as valuable tools for healthcare professionals and organizations seeking to educate and empower women to protect themselves and their partners from HPV and CC. Moreover, this approach encourages timely screening, diagnosis, and treatment when necessary. On a larger scale, this intervention has the potential to contribute to population-level prevention efforts by reducing the transmission and prevalence of HPV and related cancers.

## Conclusion

 In conclusion, the findings of this study demonstrate a significant improvement in knowledge and perceived risk of CC following the use of both the mobile app and digital booklet. The short, targeted educational content in both formats seems to have played a critical role in raising awareness and perceived risk among participants. However, cognitive barriers (such as fear), preventive self-efficacy, and perceived social support showed more pronounced improvement in the mobile app group. This suggests that mobile apps, with their varied content delivery formats, may have a stronger impact on the psychological and behavioral aspects of CC prevention. These findings underscore the effectiveness of educational interventions tailored to women’s needs, using the I-Change model, in fostering preventive behaviors related to CC. It is recommended that similar educational approaches be integrated into CC prevention programs, especially for women from low-income or less-educated backgrounds. Future research should continue to explore the impact of mobile app-based education on preventive behaviors for CC in greater depth.

## Competing Interests

 The authors have no conflict of interest to report.

## Data Availability Statement

 The datasets utilized and/or analyzed in the present study can be obtained from the corresponding author upon reasonable request.

## Ethical Approval

 This study was approved by the Ethics Committee of Tarbiat Modares University, Tehran, Iran (code number: IR.MODARES.REC.1400.049). All participants were informed about the study’s purpose, methods, and potential benefits, and written informed consent was obtained. Confidentiality and anonymity of participant data were strictly maintained throughout the study.
